# Determinants of early marriage among married women in nine high fertility sub-Saharan African countries: a multilevel analysis of recent demographic and health surveys

**DOI:** 10.1186/s12889-022-14840-z

**Published:** 2022-12-15

**Authors:** Tadele Biresaw Belachew, Wubshet Debebe Negash, Getachew Teshale Kefale, Tesfahun Zemene Tafere, Desale Bihonegn Asmamaw

**Affiliations:** 1 Department of Health Systems and Policy, Institute of Public Health, College of Medicine and Health Sciences, University of Gondar, P.O.Box: 196, Gondar, Ethiopia; 2 Department of Reproductive Health, Institute of Public Health, College of Medicine and Health Sciences, University of Gondar, Gondar, Ethiopia

**Keywords:** Early marriage, Female, Multilevel, Factors, Sub-Saharan Africa

## Abstract

**Background:**

Early marriage is global issue that seriously harms women’s personal development and rights. Regarding this, information about married women’s early marriage is inadequate in the world, including sub-Saharan Africa; therefore, this study aimed to assess the early marriage of women in the top nine highly fertile SSA countries.

**Methods:**

Data for this study was obtained from the most recent Demographic and Health Surveys. A total weighted sample of 121,077 married reproductive-age women was included. A multilevel mixed-effect binary logistic regression model was fitted to identify the significant associated factors of early marriage. As a final step, the Adjusted Odds Ratio (AOR) was used with a confidence interval of 95% in determining statistical significance.

**Results:**

Overall prevalence of early marriage was 55.11% (95% CI: 54.8, 55.4) and ranged from 28.11% in Burundi to 80.77% in Niger. The factors significantly associated with early marriage were women’s educational status; primary education (AOR = 0.39; 95% CI: 0.38, 0.41), secondary and higher (AOR = 0.1; 95% CI: 0.09, 0.11), employed (AOR = 0.73; 95% CI: 0.71, 0 .75), classified as rich wealth index level (AOR = 0.87; 95% CI: 0.85, 0.91), a number of family size ≥ 7 (AOR = 1.28; 95% CI: 1.23, 1.33), community-level poverty, (AOR = 1.28; 95% CI: 1.23, 1.33) and rural residency (AOR = 1.16;95% CI: 1.12, 1.21).

**Conclusion:**

Marriage before the age of 18 is moderately high in high-fertility countries. Therefore, the respective countries government should give due attention to access to education, and encourage the participation of women in making marriage-related decisions, especially those residing in rural areas.

## Background

Early marriage refers to a marriage that occurs before the age of 18, and in which the girl is not prepared for marriage and childbirth [1, 2]. In the world, over 700 million women are married before they are 18 years old [3]. There is a wide variation in the number of early marriages between countries and regions. According to the World Bank, the highest rates of early marriage have been reported in South Asia [4] and sub-Saharan Africa [5], where 44 and 39% of girls, respectively, were married before turning 18. Statistical data from 33 countries show that marriage trends haven’t changed much since the International Conference on Population and Development [4]. More over 19% were in East Asia and Pacific and 18% in Middle East and North Africa [5].

Early marriage has several negative consequences for women and their children in terms of health and social outcomes. These risks include depression and suicidality; compromised sexual, reproductive, and maternal health [6–10]; A higher risk of intimate partner violence [8, 11, 12]. In addition, early marriage compromises girls’ ability to attend school, leading to school withdrawals [7, 13–16]. As such, it is a public health concern that violates international human rights laws and seriously impairs the health and development of women and children [5, 9, 17–20].

Many factors contribute to the increase in early marriage, including incentives to marry young women out to relieve the economic burden on disadvantaged families [18]. Furthermore, some parents believe that marrying off their daughters to well-off families will improve their social status and protect their daughters from sexual adversity [9, 21]. Moreover, many studies in the world have identified the factors contributing to early marriage. These factors include family income, family size, educational level of the respondents, first sexual encounters by young women before 16 years old, residence, wealth status, perceived marriage age, and exposure to the media [22–28].

The issue of early marriage has been addressed in a variety of ways on a global and regional level over the past decade’s [2, 10, 29, 30]. With the prevalence of child marriage, the UN formulated Sustainable Development Goal-3 (SDG-3) aimed at contributing significantly to the health and well-being of many countries [31]. Developing young women’s potential as productive and healthy individuals is a critical part of SDG-3 [32]. Even though the above strategies have been implemented, however, the prevalence of early marriage in sub-Saharan African countries consistently high [32–35]. Although studies were conducted in specific countries like Nigeria [33] Democratic republic Congo [25] and Mali [36]. A study combining these high fertility countries (Nigeria, Gambia, Burkina Faso, Niger, Democratic Republic Congo, Mali, Chad, Angola, and Burundi) has not been conducted.

In addition the issue has not been adequately explored, and the lack of literature on it may hinder effective efforts, policies and interventions, particularly in sub-Saharan African countries. Hence, this study aimed to determine the prevalence of early marriage, as well as its determinants (both individual and community-level factors) in the top nine highly fertile sub-Saharan African countries.

## Methods

### Study settings and data source

This study utilized pooled data from the latest Demographic and Health Surveys (DHS) conducted between January 2010 and December 2018 of nine countries in SSA. Niger, Democratic Republic of Congo, Mali, Chad, Angola, Burundi, Nigeria, Gambia, and Burkina Faso were included in this study. These countries were selected because they are the top ten countries with high fertility rates in SSA with fertility rates above 5.0, a higher value than the rate of 4.44 in SSA and 2.47 worldwide [37]. One country (Somalia) with no DHS data was excluded from the analysis. The data for these countries were obtained from the official database of the DHS program, www.measuredhs.com after authorization was granted via online request by explaining the purpose of our study. We used the women record (IR file) data set and extracted the dependent and independent variables. The DHS is a nationally representative household survey that uses face-to-face interviews on a wide range of population, health, nutrition tracking, and effect assessment measures. Study participants were selected using a two-stage stratified sampling technique. Enumeration Areas (EAs) were randomly selected in the first stage, while households were selected in the second stage [38]. A total weighted sample of 121,077 reproductive-age women was included in the study (Table 1).

**Table 1:** Description of Surveys and sample size characteristics in highly fertile countries in SSA (*n* = 121,077)

Countries	Survey year	Weighted sample(n)	Weighted percentage (%)
Angola	2015	9313	7.69
Burkina Faso	2010	14,095	11.64
Burundi	2016	11,302	9.33
Chad	2014	14,779	12.21
DR Congo	2013	13,928	11.50
Gambia	2013	7270	6.00
Mali	2018	8841	7.30
Nigeria	2018	31,270	25.83
Niger	2012	10,277	8.49

### Outcome variable (v511)

The outcome variable for this study was early marriage, defined as young girls married before their 18th birthday [36, 39, 40]. It was dichotomized and coded as “yes” =1 if the age at first cohabitation among the women occurred before their 18th birthday and “no” =0 if the first marriage was at 18 years and above.

### Explanatory variables

Individual and community level independent variables were included in this study.

Individual level variables; Educational status of respondents, husband education, occupation of respondents, husband occupation,wealth status, media exposure, number of living children.

Community level variables; Community level variables included residences and some were derived from the individual level data of all community members in the primary sampling unit (PSU), which includes the community level poverty, community education, community employment and community level media exposure.

### Data analysis

For data analysis Stata version 16 software was used. To ensure the representativeness of the DHS sample and obtain reliable estimations and standard errors, data were weighted (v005/1000000) before analyzing it.

The study fitted four models: the null model with no explanatory variables, model I with individual factors, model II with community factors, and model III with both individual and community factors. As the models were nested, the Intra class Correlation Coefficient (ICC), Median Odds Ratio (MOR) and, deviance (−2LLR) values were used for model comparison and fitness, respectively. Model III was the best-fitting model due to its low deviance. In the multivariable analysis, variables with a *p*-value less than 0.2 in bivariable analysis were used. Finally, in the multivariable analysis, adjusted odds ratios with 95% confidence intervals and *p*-values less than 0.05 were utilized to identify factors of early marriage.

## Results

### Individual level factors

Out of the total respondents, 53.85% women were not attended formal education, 67.62% had no work, and 60.62% had media exposure towards early marriage. Among the participants, 44.54% had seven and above family size. With regard to their economic status, 40.42% women were from the poor wealth quintiles and 39.51% were from the rich wealth quintiles (Table 2).

**Table 2:** Individual characteristics of respondents in high fertility countries in sub-Saharan Africa (*n* = 121,076)

Variables	Categories	Frequency	Percentage (%)
Educational status of respondents	No formal education	65,201	53.85
	Primary education	27,036	22.33
	Secondary and higher	28,840	23.82
Husband education	No formal education	54,003	44.60
	Primary education	20,315	16.78
	Secondary and higher	46,758	38.62
Occupation of respondents	Employed	39,201	32.38
	Unemployed	81,876	67.62
Husband occupation	Employed	109,336	90.30
	Not employed	11,741	9.70
Wealth index	Poor	48,936	40.42
	Middle	24,307	20.08
	Rich	47,833	39.51
Media exposure	Yes	73,393	60.62
	No	47,683	39.38
Number of household members	1–3	19,177	15.84
	4–6	47,967	39.62
	≥7	53,932	44.54

### Community level factors

Of the respondents, 68.20% were rural dwellers. More than half (51.56%) of the respondents were from communities with low proportion of poverty level. Half (50.41%) of women had media exposure. Above 50% (50.64%) of participants were from communities having high proportion of community level education (Table 3).

**Table 3:** Community level characteristics of respondents in high fertility countries in sub-Saharan Africa (*n* = 121,076)

Variables	Categories	Frequency	Percentage (%)
Residence	Urban	38,498	31.80
	Rural	82,579	68.20
Community-level poverty	Low	62,428	51.56
	High	58,648	48.44
Community media exposure	Low	60,040	49.59
	High	61,036	50.41
Community employment	Low	61,903	51.13
	High	59,174	48.87
Community education	Low	56,094	49.36
	High	57,540	50.64

### Prevalence of early marriage in top nine highly fertile sub-Saharan African countries

**Fig. 1:**
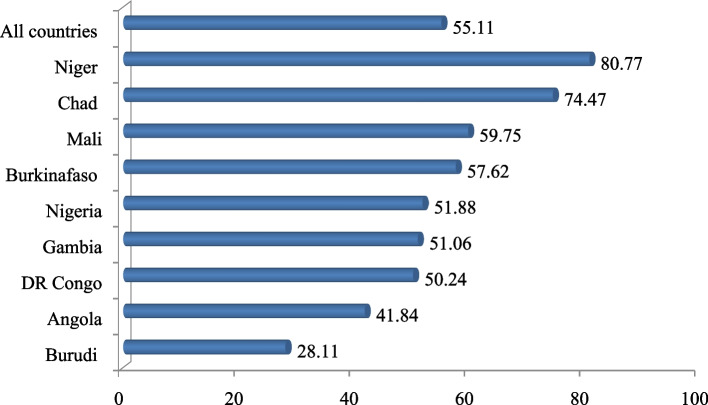
Prevalence of early marriage in top nine highly fertile SSA countries

Overall, the prevalence of early marriage in top nine highly fertile sub-Saharan African countries was 55.11% (54.8, 55.4). The prevalence of early marriage ranged from 28.11% in Burundi to 80.77% in Niger (Fig. 1).

### Factors associated with early marriage practice

Regarding individual level factors, the study found that women with a secondary or higher education were 90% less likely to be married below the age of 18 years than those who had no formal education (AOR = 0.1; 95% CI: 0.09, 0.11) and those women with primary education were 61% less likely to be married below the age of 18 years compared to women who have never had formal education (AOR = 0.39; 95% CI: 0.38, 0.41). Women who were working were 27% less likely to be married below the age of 18 years compared to those who had no working (AOR = 0.73; 95% CI: 0.71, 0 .75). The odds of being married below at 18 years in the rich level were 13% less likely compared to women who live in poverty (AOR = 0.87; 95% CI: 0.85, 0.91). The likelihood of women’s early marriage was high among women who had ≥7 families size (AOR = 1.28; 95% CI: 1.23, 1.33) compared to 1–3 families size.

About the community level factors, married women classified as high Community level poverty were more likely to have early marriage (AOR = 1.09; 95% CI: 1.01, 1.17) than low Community level poverty. In addition the odds of being married below the age of 18 in rural area were 1.16 more likely than living in an urban area (AOR = 1.16; 95% CI: 1.12, 1.21) (Table 4).

**Table 4:** Multivariable analyses for factors affecting early marriage practice **(***n* = 121,076)

Variables	Model 0	Model 1 AOR (95% CI)	Model 2 AOR (95%CI)	Model 3 AOR (95%CI)
**Individual level Characteristics**				
**Educational status of the respondents**				
No formal education		1		1
Primary education		0.09 (0.08,0.10)		**0.39 (0.38, 0.41)**
Secondary and higher		0.37 (0.356, 0.39)		**0.01 (0.09,0.11)**
**Husband education**				
No formal education		1		1
Primary education		0.84 (0.81, 0.87)		0.86 (0.83, 1.10)
Secondary and higher		0.80 (0.76, 0.84)		0.85 (0.80, 1.89)
**Occupation of respondents**				
Had not work		1		1
Had Work		0.74 (0.72, 0 .76)		**0.73 (0.71, 0 .75)**
**Husband Occupation**				
Had not work		1		1
Had Work		1.09 (1.05, 1.14)		1.08 (0.75, 1.13)
**Wealth index**				
Poor		1		1
Middle		0.95 (0.92, 0 .99)		0.97 (0.94, 1.01)
Rich		0.81 (0.78, 0.83)		**0.87 (0.85, 0.91)**
**Media exposure**				
No		1		1
Yes		0.91 (0.88, 0.93)		0.93 (0.91, 1 .08)
**Family size**				
1–3		1		1
4–6		1.05 (1.01, 1.09)		1.03 (0.99, 1.07)
≥ 7		1.32 (1.27, 1.37)		**1.28 (1.23, 1.33)**
**Community level variables**				
**Community level poverty**				
Low			1	1
High			1.22 (1.10, 1.34)	**1.09 (1.01, 1.17)**
**Community level media exposure**				
Low			1	1
High			0.76 (0.69, 0 .84)	0.85 (0.79, 1.19)
**Residency**				
Urban			1	1
Rural			1.81 (1.75, 1.86)	**1.16 (1.12, 1.21)**
**Community level education**				
Low			1	1
High			0.63 (0.57, 0 .70)	0.83 (0.77, 1.09)
**Community level employment**				
Low			1	1
High			1.04 (0.96, 1.13)	1.21 (0.98, 1.29**)**
**Random effect results**				
Variance (%)	52.5	31.6	27.95	16.3
ICC (%)	24.4	17.8	18.8	14.7
MOR	18.7	14.5	13.67	10.4
PCV	Ref	39	46.76	68.95
Deviance(−2LLR)	159,670	143,448	149,568	136,512

*Statistically significant at *p*-value< 0.05,

AOR Adjusted Odds Ratio, COR Crude Odds Ratio

Null model: adjusted for individual-level characteristics,

Model 2: Adjusted for community-level characteristics,

Model 3: adjusted for both individual and community-level characteristics

## Discussion

This study revealed the prevalence of early marriage in the top nine highly fertile sub-Saharan African countries was 55.11% (95% CI: 54.8, 55.4). This finding is in line with previous studies in Sub-Saharan Africa [32]. This finding is higher than a study conducted in Injibara, Ethiopia [24]. Moreover, the finding is also higher than in studies conducted in Sudan [41], India [26], and Roma of Serbia [42]. This prevalence, however, is lower than that study conducted in east Gojjam, Ethiopia [43], Amhara Regional State, Ethiopia [44], Ethiopia [44], and a study conducted in Bangladesh [45]. This discrepancy may result from the smaller sample size in the previous studies than in the current study.

The study revealed that women with primary education and secondary and above education were 61 and 90% less likely to be married below the age of 18 years compared to those with no formal education respectively. This study backs up research from Ethiopia that found that a woman’s educational degree is a strong predictor of early marriage [46, 47]. Moreover, other studies conducted in Malawi [42] and Western Uganda [42] revealed that women’s education level was an independent predictor of early marriage [20, 48]. This might be due to the fact that education helps people to know about their rights and enables them to make informed decisions when it comes to marriage [3, 49, 50].

Moreover, this study found that early marriage was lower among women who had work compared to women who had no work. Comparable findings were found in a study conducted in Gambia [51]. Additionally, Singh and Vennam [52] reported that girls who were unemployed or working in their families were more likely to marry at a younger age than those who were working, particularly those in the service industries. The odds of being married below the age of 18 in the rich level were 13% less likely compared to those women in the poor level. This result is consistent with two studies conducted in Ethiopia [47, 53] and a study done in India [54]. This might be justified by the poorest families preferring early marriage to generate more income from male family [43]. This is also supported by another study conducted in Ethiopia revealed that low economic status is one of the predisposing factors for early marriage [46, 55, 56].

Our study found that women from large-sized families were more likely to marry than women from small families. This finding is consistent with studies conducted in Sudan [41] and Ethiopia [24]. The reason could be that parents with large families use child marriage as a means of receiving bride costs, reduce their family size, and improve their financial resources [24]. According to research conducted in West and Central Africa, some rural families consider girls not only a source of wealth, but also a way to increase the family’s social status and prestige [57, 58].

In this study, the odds of early marriage among rural women were 1.16 higher compared to that of urban women. The findings of this study are similar to those from Sudan [41], Bangladesh [59], and Serbia [42]. It may be because women in rural areas may not be aware of the health, educational, and economic consequences of early marriage [55, 60]. Furthermore, they are unsure of what to do when their parents or guardians violate their human rights [55, 60, 61]. Therefore, women living in rural areas have a higher risk of early marriage than those living in urban areas.

Teenagers who live in communities with a higher proportion of poor were more likely to marry early than teenagers who live in communities with a lower proportion of poverty. This is consistent with other studies in SSA [62] and Philippines [63]. This might be due to teenagers who live in communities with poor wealth status having poor access to education and are faced with the problem of early marriage.

The study’s strength was the use of nationally representative survey data sets from large countries. Due to the cross-sectional nature of the data, this study may not demonstrate a causality and effect relationship. In addition, the dataset lacks variables such as cultural norms, behavioral patterns and social norms, which have a significant impact early marriage.

## Conclusion

The overall prevalence of early marriage among married reproductive-age women in the top nine highly fertile sub-Saharan African countries is high. Rural residence, non-formal education, wealth index, large family size, and high community-level poverty, were the independent predictors of early marriage in the top nine highly fertile sub-Saharan African countries.

Therefore, the respective countries governments should give due attention to access to education and encourage women’s decision-making power at the age of marriage particularly in rural areas of the region. Moreover, each country government should encourage women to participate in small-scale entrepreneurship to maximize their economic status.

## Data Availability

Data for this study were sourced from Demographic and Health surveys (DHS), which are freely available online at (https://dhsprogram.com).
